# A Novel Two-Component Bacteriocin, Acidicin P, and Its Key Residues for Inhibiting Listeria monocytogenes by Targeting the Cell Membrane

**DOI:** 10.1128/spectrum.05210-22

**Published:** 2023-06-08

**Authors:** Tianqi Xia, Kunling Teng, Yayong Liu, Yaoqi Guo, Fuqing Huang, Muhammad Tahir, Tianwei Wang, Jin Zhong

**Affiliations:** a State Key Laboratory of Microbial Resources, Institute of Microbiology, Chinese Academy of Sciences, Beijing, China; b School of Life Science, University of Chinese Academy of Sciences, Beijing, China; University of California Davis

**Keywords:** bacteriocin, acidicin P, *Listeria monocytogenes*, key residues, antimicrobial mechanism

## Abstract

Listeria monocytogenes is an important pathogen which easily contaminates food and causes fatal systemic infections in human. Bacteriocins have received much attention regarding their natural methods of controlling health-related pathogens. Here, we investigated and characterized a novel two-component bacteriocin named acidicin P from Pediococcus acidilactici LAC5-17. Acidicin P showed obvious antimicrobial activity to L. monocytogenes. Through a sequence similarity network analysis for two-component bacteriocin precursors mined in the RefSeq database, acidicin P was observed to belong to an unusual group of two-component bacteriocins. Acidicin P contains two peptides designated Adpα and Adpβ which are assessed to interact with each other and form a helical dimer structure which can be inserted into the lipid bilayer of target cell membrane. We demonstrate that A5, N7, and G9 in the A_5_xxxG_9_ motif of Adpα and S16, R19, and G20 in the S_16_xxxG_20_ motif of Adpβ played crucial roles in stabilizing the helix-helix interaction of Adpα and Adpβ and were essential for the antilisterial activity of acidicin P by site-directed mutagenesis. A positive residue, R14, in Adpα and a negative residue, D12, in Adpβ are also important for acidicin P to fight against L. monocytogenes. These key residues are supposed to form hydrogen bonding, which is crucial for the interaction of Adpα and Adpβ. Furthermore, acidicin P induces severe permeabilization and depolarization of the cytoplasmic membrane and causes dramatic changes in L. monocytogenes cell morphology and ultrastructure. Acidicin P has the potential to be applied to inhibit L. monocytogenes efficiently both in the food industry and medical treatments.

**IMPORTANCE** L. monocytogenes can cause widespread food contamination and severe human listeriosis, which amount to a large proportion of the public health and economic burdens. Today, L. monocytogenes is usually treated with chemical compounds in the food industry or antibiotics for human listeriosis. Natural and safe antilisterial agents are urgently required. Bacteriocins are natural antimicrobial peptides that have comparable narrow antimicrobial spectra and are attractive potentials for precision therapy for pathogen infection. In this work, we discover a novel two-component bacteriocin designated acidicin P, which shows obvious antilisterial activity. We also identify the key residues in both peptides of acidicin P and demonstrate that acidicin P is inserted into the target cell membrane and disrupts the cell envelop to inhibit the growth of L. monocytogenes. We believe that acidicin P is a promising lead for further development as an antilisterial drug.

## INTRODUCTION

Foodborne disease (FBD) is a constant threat to public health and has gained increasing attention worldwide. The food contaminated by pathogens during processing is one of the major causes of FBD (1). The World Health Organization (WHO) reported that foodborne pathogens caused 600 million foodborne illnesses and 420,000 deaths in 2010 (1). Listeria monocytogenes is one of the most common microorganisms causing foodborne diseases. It possesses internalin A (InlA) and InlB for epithelial cell invasion, *Listeria* adhesion protein (LAP) for gut barrier crossing (2), flagella involved in biofilm formation and motility (3), hemolysin listeriolysin O for cell lysing (4), and the virulence regulatory factor *prfA* (5) and therefore has a relatively high pathogenicity. It can cause listeriosis among immunocompromised individuals, such as pregnant women, the elderly, infants, and AIDS patients who eat or touch contaminated food (6). The U.S. Centers for Disease Control and Prevention (CDC) reported that the hospitalization rate of listeriosis is 94%, while the fatality rate is about 20 to 30%, even with adequate antibiotic treatment (7). Moreover, L. monocytogenes can form biofilm on food-contact surfaces and the food production environment, threatening public food safety, which makes it difficult to control (8). Hence, a strategy to effectively control L. monocytogenes is urgently required. Natural and safe antilisterial agents are in considerable demand for both the food industry and medical care.

Ribosomally synthesized antimicrobial peptides, bacteriocins, produced by food-grade lactic acid bacteria (LAB) generally recognized as safe are attractive options to control foodborne pathogens. They have favorable properties, including stability, low toxicity, the availability of both broad and narrow antibacterial spectra, the possibility of production by probiotics, and easy bioengineering (9). These properties make bacteriocins potentially useful for the bio-preservation of meat, dairy products, egg products, fermented vegetables, and so on. Moreover, they can be hydrolyzed by enzymes in the gastrointestinal tract during digestion which causes less adverse effects to human health (10).

Two-component bacteriocins contain two different individual peptides interacting with each other and functioning as one unit to exert optimal activity (11). Lactococcin G (LcnG-α and LcnG-β) was the first two-component bacteriocin and was isolated in 1992 (12). Since then, increasing numbers of members have been characterized due to their activities on controlling several pathogens, e.g., Staphylococcus spp., *Clostridium* spp., Salmonella enterica serovar Typhimurium, Escherichia coli, and L. monocytogenes (13). The conserved GxxxG-motif (x represents any three residues) and GxxxG-like motif (in which glycine residues are replaced by alanine or serine) are the general features of sequences for two-component bacteriocins. These motifs may promote proximity between the two complementary peptides via hydrogen bonds or van der Waals interactions. Furthermore, the two peptides can form multiple parallel or antiparallel transmembrane dimers upon different motifs to target sensitive cells (13). However, not all conserved motifs in the sequence participate in the interaction of the two peptides and are important for antimicrobial activity. Moreover, how the conserved motifs mediate the interactions between two complementary peptides and the formation of the transmembrane helical dimer to fight against target bacteria is not fully understood.

In our previous study, a biosynthetic gene cluster of a novel two-component bacteriocin was identified in Pediococcus acidilactici LAC 5-17 isolated from Chinese spicy cabbage using a metagenomic mining of isolates population (MMIP) method (14). It is the first biosynthetic gene cluster of a two-component bacteriocin found in *P. acidilactici*. Here, we obtained this two-component bacteriocin and designated it acidicin P. We found that acidicin P showed obvious antimicrobial activity against L. monocytogenes. The sequence similarity network (SSN) analysis of two-component bacteriocin precursors mined in the RefSeq database indicated that acidicin P belonged to an unusual group of two-component bacteriocins. We investigated the binding of the two peptides of acidicin P and the key residues in each peptide for their interaction. We also found that acidicin P could be inserted into the cell membrane of L. monocytogenes and cause severe permeabilization and depolarization of the membrane.

## RESULTS

### Acidicin P belongs to an unusual group of two-component bacteriocins.

**FIG 1:**
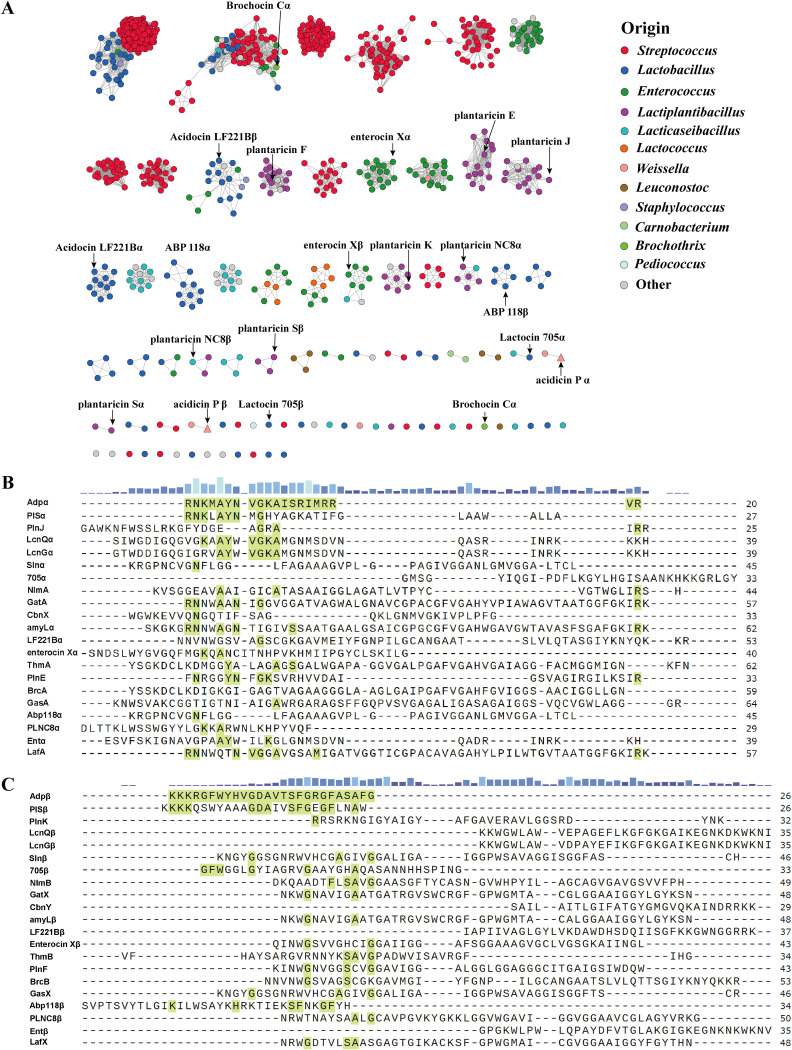
The bioinformatic analysis of two-component bacteriocins and acidicin P. (A) Precursor peptides sequence similarity network (SSN) of two-component bacteriocins. Nodes represent amino acid sequences and are colored according to the origin. The reported bacteriocins with activity against pathogens or anti-inflammatory activity were annotated in the SSN. Triangles represent two peptides of acidicin P. (B) Sequence similarity among Adpα with α peptide of other two-component bacteriocins. The sequence of Adpα is shown in green. (C) Sequence similarity among Adpβ with β peptide of other two-component bacteriocins. The sequence of Adpβ is shown in green.

PSI-BLAST analysis was performed to investigate the putative two-component bacteriocin precursor peptide sequences which are used to create the SSN. In the SSN (Fig. 1A), each node represents a unique peptide sequence, and the edge is drawn between two nodes sharing a similarity. In total, 801 precursor peptide sequences were constructed to visualize diversity and distribution status, and the related peptides were grouped together. Gene clusters of the two-component bacteriocins are widely distributed in lactic acid bacteria, such as Streptococcus, *Lactobacillus*, *Enterococcus*, *Lactiplantibacillus*, *Lacticaseibacillus*, and so on. The reported two-component bacteriocins were labeled (Table S1). The biosynthetic gene cluster (BGC) of acidicin P was discovered by *in silico* analysis in the metagenomic sequences of the microbial isolate population from Chinese spicy cabbage using the MMIP method (14) and was labeled on the SSN. As shown in Fig. 1A, acidicin P was grouped into a small family of two-component bacteriocins which has never been reported. The sequence features of Adpα (Fig. 1B) and Adpβ (Fig. 1C) were varied from other two-component bacteriocins with lower similarity. Therefore, acidicin P was an unusual and underinvestigated two-component bacteriocin.

### Biosynthetic gene cluster, amino acid sequences and molecular weight of acidicin P.

**FIG 2:**
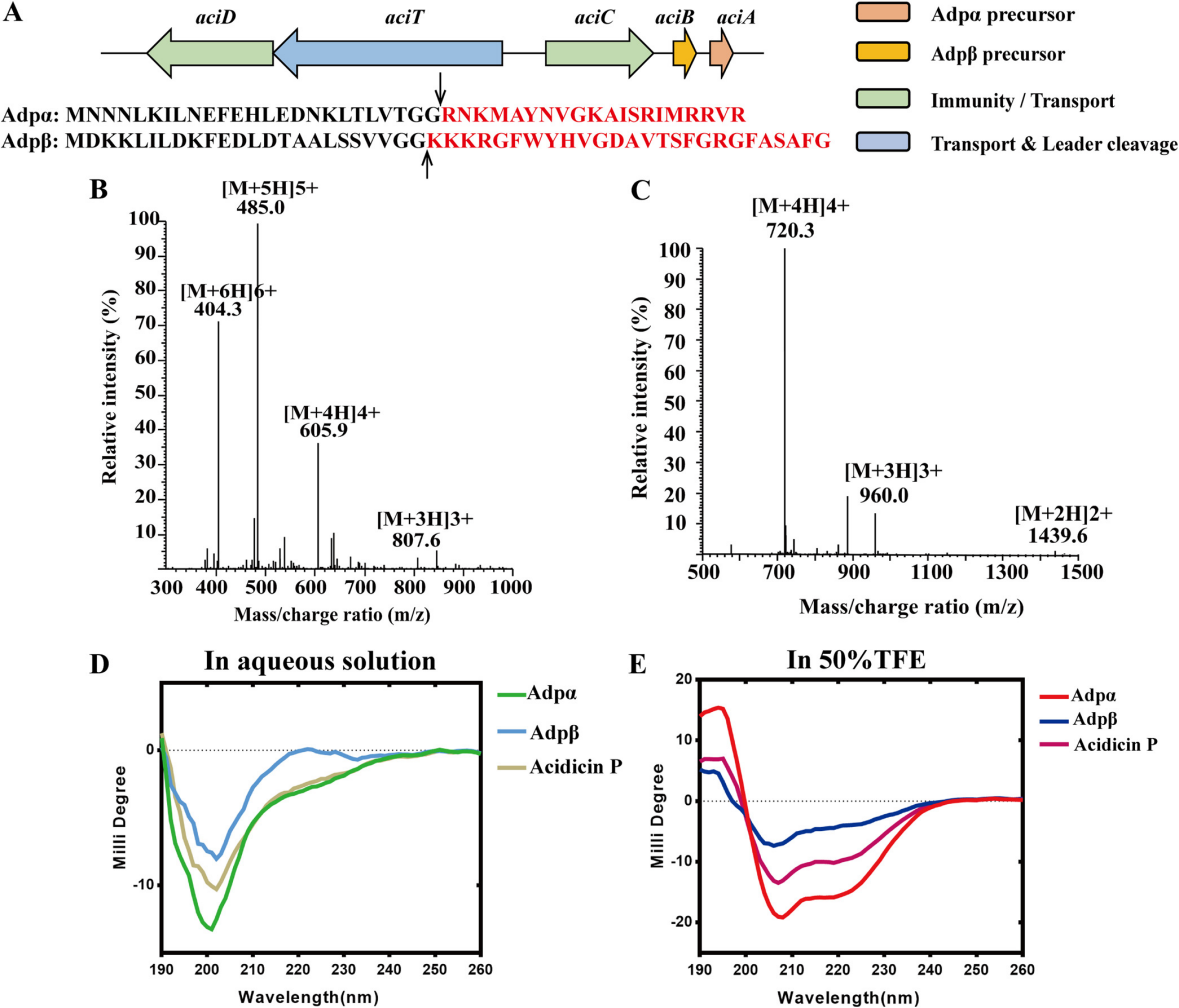
Characterization and secondary structure of acidicin P. (A) Biosynthetic gene cluster and sequences of acidicin P. *aciD* and *aciC* are two genes encoding putative immunity proteins. *aciT* is an ABC transporter and leader cleavage protease-encoding gene. *aciA* is the precursor gene of Adpα, and *aciB* is the precursor gene of Adpβ. The two sequences were the precursor sequence of Adpα and Adpβ, individually. The black arrows indicate the predicted cleavage sites. (B and C) Molecular weight of Adpα and Adpβ, respectively. (D) Circular dichroism (CD) spectroscopy of Adpα, Adpβ, and acidicin P in aqueous solution. (E) CD spectroscopy of Adpα, Adpβ, and acidicin P in 50% TFE.

The producer *P. acidilactici* LAC5-17 was obtained, and the BGC of acidicin P was analyzed. The BGC of acidicin P comprises five genes, *aciD*, *aciT*, *aciC*, *aciB*, and *aciA* (Fig. 2A), which encode two putative precursors, two putative immunity proteins (AdpC and AdpD), one ABC transporter, and a leader cleavage protease (AdpT). Both acidicin P precursor peptides contain leader sequences with a double-glycine cleavage site that are predicted to be processed by AdpT to produce mature peptides with 20 and 26 amino acids, and we designated them Adpα and Adpβ, respectively. Adpα and Adpβ are predicted to be transferred across the membrane by AdpT to perform their functions. The immunity proteins AdpC and AdpD are predicted to protect the producer from being killed by its own bacteriocin. However, we did not detect acidicin P in the cell-free culture supernatant of *P. acidilactici* LAC5-17. Finally, we obtained Adpα and Adpβ by solid-phase peptide synthesis. Our results showed that the molecular weight (MW) of Adpα and Adpβ was 2,420.0 and 2,877.2 Da (Fig. 2B, C), respectively, corresponding to their theoretical MW of 2,419.94 and 2,877.26 Da.

### Secondary structure of acidicin P.

The secondary structures of Adpα, Adpβ, and acidicin P (Adpα and Adpβ combined in equal amounts) were checked by circular dichroism (CD) spectroscopy. The CD spectra of Adpα, Adpβ, and acidicin P in aqueous solution all have characteristics of nonstructured conformations (Fig. 2D). Trifluoroethanol (TFE) induced a typical α-helical structure which displays negative peaks at 222 nm and 208 nm, respectively, and a positive peak at 193 nm (Fig. 2E). At 50% TFE, calculated helicities were 24.9%, 16.5%, and 20.0% for Adpα, Adpβ, and acidicin P, respectively. Premixing Adpα and Adpβ before CD analysis induced helicity which was higher than that of Adpβ but lower than that of Adpα. These results illustrate that the helical structure occurred when the peptides were individually exposed to a membrane-like environment, and additional structure appeared when complementary peptides were premixed.

### Antimicrobial activity of acidicin P.

**FIG 3:**
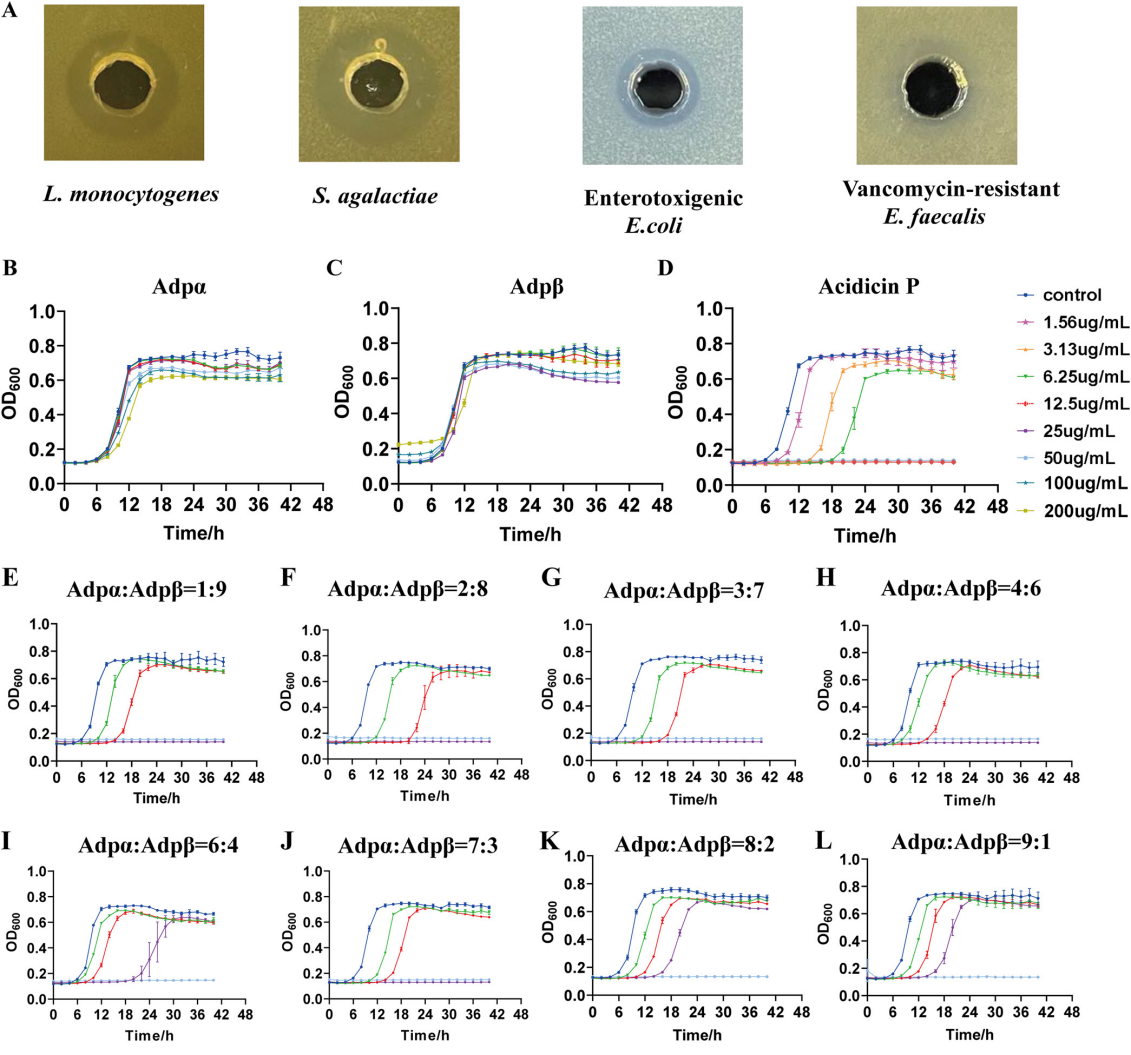
Antimicrobial activity of acidicin P. (A) The antimicrobial spectrum of acidicin P. (B) Growth curves of Listeria monocytogenes treated by gradual concentrations of Adpα, showing the time-kill kinetics of individual Adpα against Listeria monocytogenes. (C) Growth curves of L. monocytogenes treated by gradual concentrations of Adpβ, showing the time-kill kinetics of individual Adpβ against L. monocytogenes. (D) Growth curves of L. monocytogenes treated by gradual concentrations of acidicin P (complementary peptides combined with equal amounts), showing the time-kill kinetics of acidicin P against L. monocytogenes. (E to L) Growth curves of L. monocytogenes treated by gradual concentrations of acidicin P with complementary peptides combined with different ratios, showing their time-kill kinetics against L. monocytogenes. The colors of the lines indicate the total concentrations of the mixed peptides.

As observed from the agar well diffusion assay (Fig. 3A), acidicin P (Adpα and Adpβ with a concentration ratio of 1:1) had a broad antibacterial spectrum inhibiting the indicator strains, such as Streptococcus agalactiae 10465, L. monocytogenes 1.10753, enterotoxigenic Escherichia coli K88, and vancomycin-resistant Enterococcus faecalis V583. L. monocytogenes is an important foodborne pathogen and is obviously sensitive to acidicin P. In order to make sure whether Adpα or Adpβ is necessary for its antimicrobial activity, the growth of L. monocytogenes treated with Adpα or Adpβ with different ratios was examined. As shown in Fig. 3B and C, the growth rate of L. monocytogenes was not affected by Adpα or Adpβ individually at any concentration, highlighting that both Adpα and Adpβ were necessary for the antimicrobial activity of acidicin P. When Adpα was present in excess (Fig. 3I, K, and L), with the increased ratio of Adpβ (Adpα:Adpβ ratio of 9:1, 8:2, and 6:4), the growth of L. monocytogenes was inhibited at 50 μg/mL, except for the ratio of 7:3 (25 μg/mL). Similarly, when Adpβ was present in excess (Fig. 3E to H), with the increased ratio of Adpα (Adpα:Adpβ ration of 1:9, 2:8, 3:7, and 4:6), the growth of L. monocytogenes was inhibited at 25 μg/mL. The lowest MIC (12.5 μg/mL) was obtained when the volume ratio of Adpα and Adpβ was 1:1 (Fig. 3D; Table 1). These findings suggest that equivalent amounts of Adpα and Adpβ have maximum inhibitory activity against L. monocytogenes, and Adpα and Adpβ might interact with each other to exert their activities.

**TABLE 1:** Synergistic antibacterial effect of Adpα and Adpβ against L. monocytogenes^*a*^

Indicator	Ratio α:β	MIC (g/mL)	MIC (μM/L)
Listeria monocytogenes 1.10753	1:0	**/**	**/**
	1:9	25	4.72
	2:8	25	4.72
	3:7	25	4.72
	4:6	25	4.72
	1:1	12.5	2.36
	6:4	50	9.44
	7:3	25	4.72
	8:2	50	9.44
	9:1	50	9.44
	0:1	**/**	**/**

a /, No antimicrobial activity observed in the range of 1.56 to 200 μg/mL acidicin P.

### Three-dimensional structure and molecular dynamics (MD) simulation of acidicin P.

**FIG 4:**
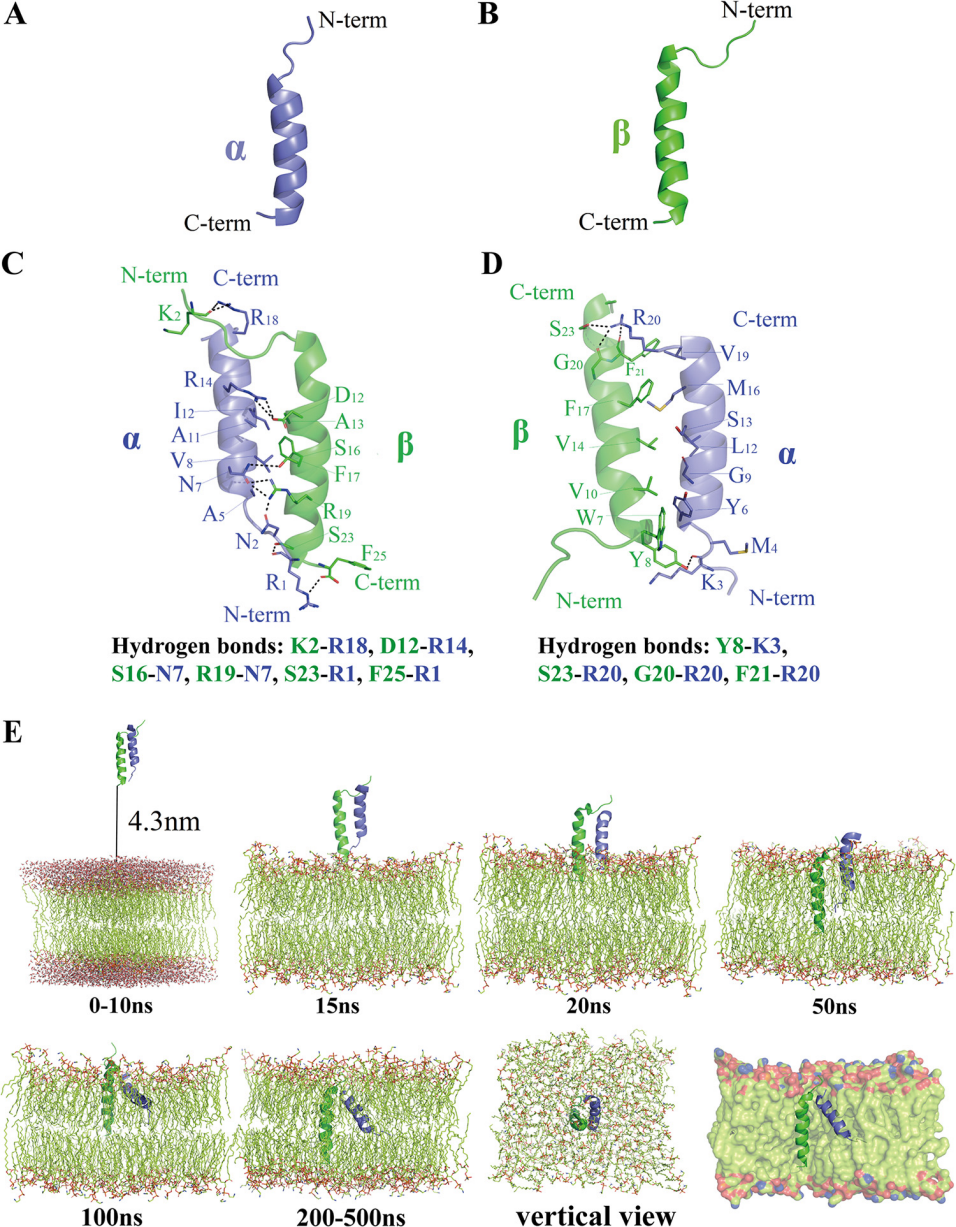
Three-dimensional structure and molecular dynamics (MD) simulation of acidicin P. (A) α-Helix structure of the Adpα. (B) α-Helix structure of the Adpβ. (C) Antiparallel orientation of the two peptides. (D) Parallel orientation of the two peptides. (E) Trajectories of the two peptides being inserted into the lipid bilayer in antiparallel orientation. C-term indicates C terminus, N-term indicates N terminus.

The simulated three-dimensional structures of Adpα and Adpβ showed that both peptides are helical (Fig. 4A and B). Adpα, a 20-residue peptide, is composed of an α-helix that extends from residues 4 to 19. Adpβ, a 26-mer, has a C-terminal α-helix extending from residues 8 to 24 and an unstructured N-terminal region. Specific peptide-peptide interaction formed by Adpα and Adpβ through a parallel or an antiparallel orientation were constructed. Fig. 4C shows 6 stabilizing backbone hydrogen bonds, K2 (Adpα) and R18 (Adpβ), D12 (Adpα) and R14 (Adpβ), S16 (Adpα) and N7(Adpβ), R19 (Adpα) and N7(Adpβ), S23 (Adpα) and R1 (Adpβ), and F25 (Adpα) and R1 (Adpβ), in the antiparallel orientation model. In the parallel orientation model (Fig. 4D), there are only 4 hydrogen bonds: Y8 (Adpα) and K3 (Adpβ), S23 (Adpα) and R20 (β), G20 (Adpα) and R20 (Adpβ), and F21 (Adpα) and R20 (Adpβ). It seems that Adpα and Adpβ form a more stable structure in the antiparallel orientation with more hydrogen bonds. We supposed that Adpα and Adpβ form heterologous dimers in an antiparallel orientation to exert their antimicrobial activities.

In order to further illustrate how acidicin P fights against the target cell, the interaction between the heterologous dimer formed by Adpα and Adpβ with antiparallel orientation and a cell membrane lipid bilayer were simulated, and the trajectories were examined (Fig. 4E). The antiparallel dimer was placed above the lipid bilayer model with a distance of about 4.3 nm at 0 ns. The dimer approached the lipid bilayer model at 15 ns. Subsequently, the peptides are inserted into the upper bilayer gradually and completely inserted into the bilayer for the termini at 200 ns. The peptides stayed in the bilayer and maintained a stable construction at 200 to 500 ns. The simulation indicates that acidicin P may be inserted into the phospholipid bilayers to disrupt the cell membrane.

### Structure-activity relationship of acidicin P.

**FIG 5:**
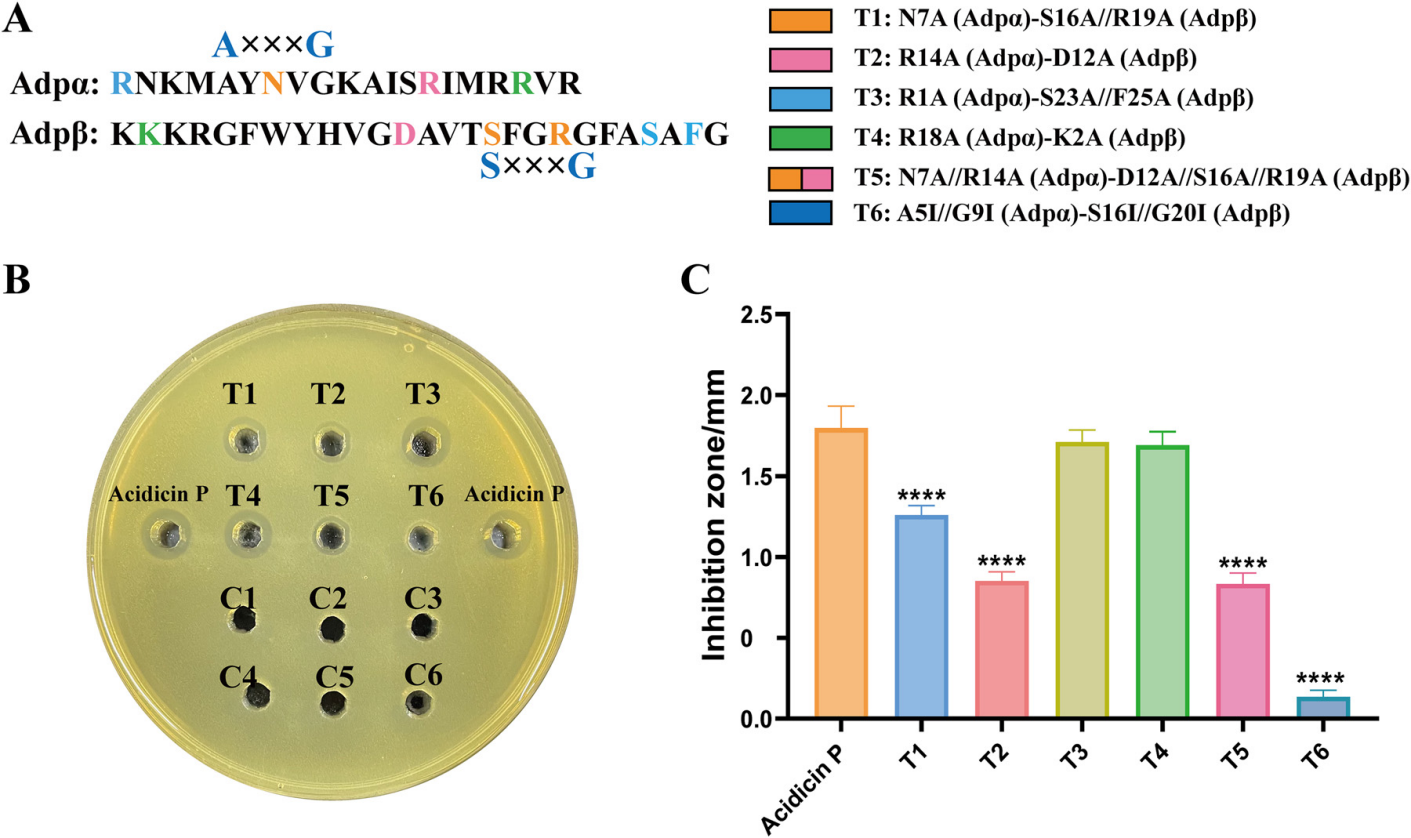
Structure-activity relationships of acidicin P. (A) Mutation grouping. Different colors in Adpα and Adpβ represent the different mutation groups used to investigate the effects of the hydron bonds and GxxxG-like motif on activity. (B) Inhibition activity of the mutations and the wild acidicin P against Listeria monocytogenes. T1-6, the mutations; C1-6, the negative control corresponding to T1-6. (C) The diameter of the transparent circle. The diameter of the hole was 5.29 mm is represented by *x*. The diameter of the transparent zone is represented by *y*. The inhibition zone (*z*) was calculated based on the following formula: *z* = *y* – *x* / 2. ****, *P *< 0.0001 versus the acidicin P group.

Either Adpα or Adpβ contains a conserved GxxxG-like motif. Adpα contains an A_5_xxxG_9_ motif and Adpβ contains an S_16_xxxG_20_ motif (Fig. 5A). In order to investigate the role of hydrogen bonds and the GxxxG-like motif in mediating the interaction of Adpα and Adpβ, site-directed mutagenesis was carried out, and their antimicrobial activities were checked (Fig. 5A; Table 2). The antimicrobial activities of mutant variants N7A (Adpα)-S16A//R19A (Adpβ), R14A (Adpα)-D12A (Adpβ), N7A//R14A (Adpα)-D12A//S16A//R19A (Adpβ), and A5I//G9I (Adpα)-S16I//G20I (Adpβ) were significantly decreased compared to the wild type, while those of R1A (Adpα)-D12A (Adpβ) and R18A (Adpα)-K2A (Adpβ) had no detrimental effect on the antimicrobial activity (Fig. 5B and C). Moreover, no bactericidal zone was observed in variant A5I//G9I (Adpα)-S16I//G20I (Adpβ). Our results showed that the mutations which destroy the formation of hydron bonds decreased the antimicrobial activity of acidicin P.

**TABLE 2:** Amino acid sequences of the mutations^*a*^

Name	Amino acid sequence		Amino acid substitution	
	Adpα	Adpβ	Adpα	Adpβ
Wild type	RNKMAYNVGKAISRIMRRVR	KKKRGFWYHVGDAVTSFGRGFASAFG	**/**	**/**
T1	RNKMAYAVGKAISRIMRRVR	KKKRGFWYHVGDAVTAFGAGFASAFG	N7A	S16A, R19A
T2	RNKMAYNVGKAISAIMRRVR	KKKRGFWYHVGAAVTSFGRGFASAFG	R14A	D12A
T3	ANKMAYNVGKAISRIMRRVR	KKKRGFWYHVGDAVTSFGRGFAAAAG	R1A	S23A, F25A
T4	RNKMAYNVGKAISRIMRAVR	KAKRGFWYHVGDAVTSFGRGFASAFG	R18A	K2A
T5	RNKMAYAVGKAISAIMRRVR	KKKRGFWYHVGAAVTAFGAGFASAFG	D12A, R14A	S16A, R19A, N7A
T6	RNKMIYNVIKAISRIMRRVR	KKKRGFWYHVGDAVTIFGRIFASAFG	A5I, G9I	S16I, G20I

a The residues were substituted with alanine without changing the structure of the peptides. The substituted residues are underlined. / means the amino acid sequence of peptide has not changed.

### Acidicin P destroyed the morphological and intracellular structure of L. monocytogenes.

**FIG 6:**
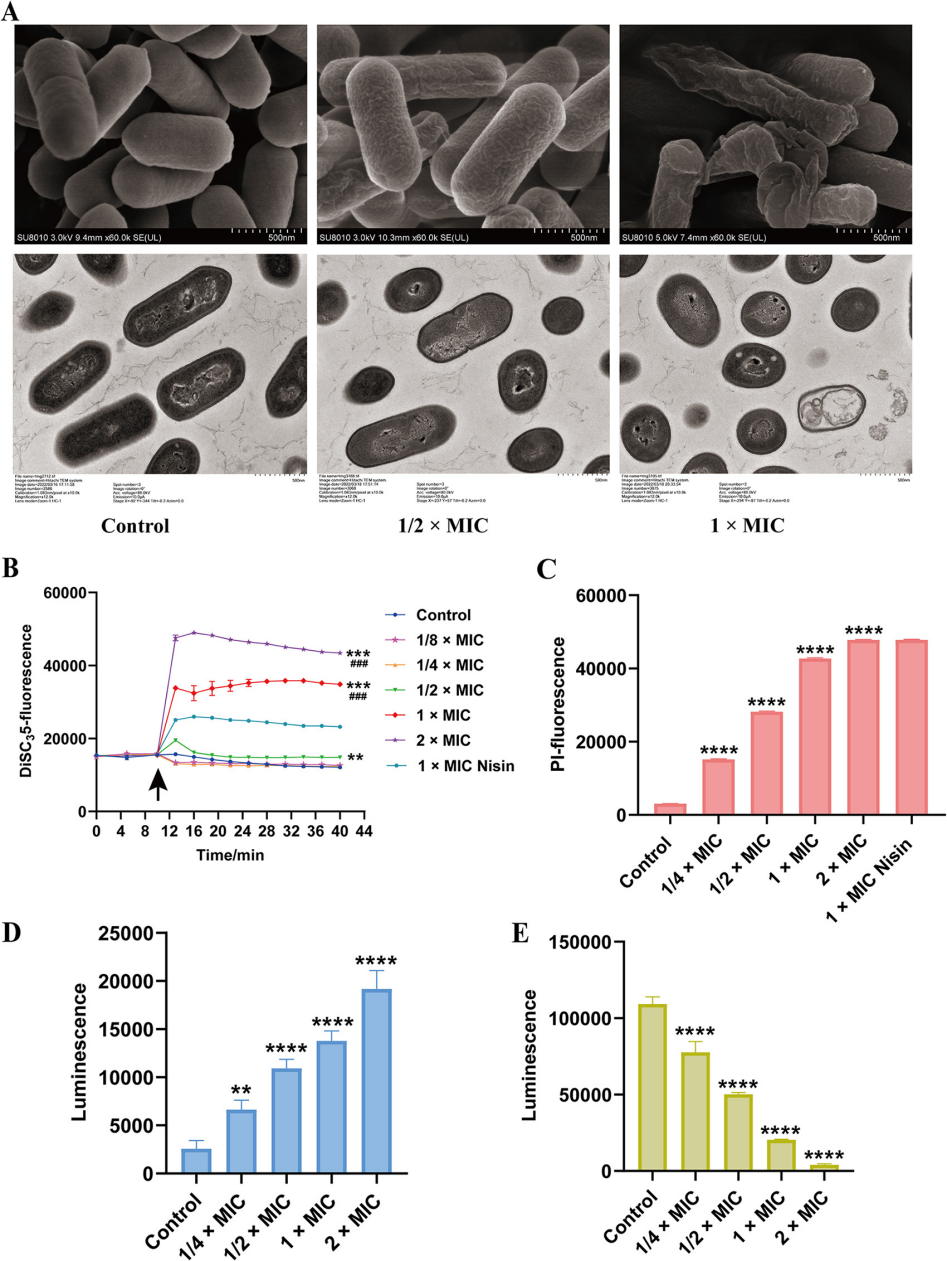
The mode of action of acidicin P against Listeria monocytogenes. (A) SEM (top) and TEM (bottom) images of L. monocytogenes treated with 1/2× or 1× MIC acidicin P. The control was treated with PBS. (B) Depolarization of the L. monocytogenes cell membrane treated with acidicin P measured by the membrane potential-sensitive fluorescent probe DiSC_3_(5). Nisin (1× MIC) was used as a positive control. The group treated with PBS was used as a negative control. The arrow indicates the time point of drug addition. **, *P *< 0.01; ***, *P *< 0.001 versus the control group. ###, *P* < 0.001 versus the nisin group. (C) Permeabilization of the L. monocytogenes cell membrane treated with acidicin P measured by fluorescent probe PI. PI was used to measure the fluorescence. Nisin (1× MIC) was used as a positive control. The control group was treated with PBS. ****, *P *< 0.0001 versus the control group. (D and E) Levels of extracellular ATP (D) and intracellular ATP (E) of L. monocytogenes treated with acidicin P. **, *P *< 0.01; ****, *P *< 0.0001 versus the control group.

Scanning electron microscopy (SEM) was performed to observe the surface morphological changes of L. monocytogenes after acidicin P treatment (Fig. 6A, top). The untreated L. monocytogenes exhibited a smooth surface and well-defined rod shape. Under 6.25 μg/mL acidicin P treatment, the L. monocytogenes cells maintained typical dimensions but had a collapsed and shrunken surface. The L. monocytogenes cells showed severe morphological destruction with evidently irregular wrinkles under treatment of 12.5 μg/mL acidicin P. Transmission electron microscopy (TEM) was performed to observe the intracellular structural changes of L. monocytogenes cells after acidicin P treatment (Fig. 6A, bottom). The L. monocytogenes cells without treatment displayed intact cell walls and cytoplasmic membranes with dense cytoplasm uniformly distributed. After being treated with 6.25 μg/mL acidicin P, the L. monocytogenes cells showed a thinner layer of intracellular cytoplasmic constituents. Under treatment with 12.5 μg/mL acidicin P, the cytoplasmic constituents leaked to the environment, and vacuolization of cell was observed. These results indicate that acidicin P induced severe damage in both the intracellular tissue and cellular structure of L. monocytogenes, and the deformation occurred in a concentration-dependent manner.

### Acidicin P disrupted PMF and increased membrane permeability of L. monocytogenes.

We analyzed the proton motive force (PMF) of L. monocytogenes cells treated with acidicin P using 3,3-dipropylthiadicarbocyanine iodide [DISC_3_(5)]. Nisin was used as a positive control in our study. As shown in Fig. 6B, the addition of nisin resulted in an increase in the fluorescence, while the negative control group showed no effect on the fluorescence. The 12.5-μg/mL (1× MIC) and 25-μg/mL (2× MIC) acidicin P treatments caused a dramatic increase in the fluorescence which was stronger than that of the nisin-treated group. Acidicin P at 6.25 μg/mL (1/2× MIC) caused an increase in the fluorescence between 10 and 13 min and then a decrease for a stable value, while 1.56 μg/mL (1/8× MIC) and 3.13 μg/mL (1/4× MIC) acidicin P caused a decrease in the fluorescence between 10 and 13 min and then an increase for a stable value. These results indicated that lower concentrations of acidicin P (<6.25 μg/mL) disrupted the PMF transiently, while higher concentrations (12.5 to 25 μg/mL) of acidicin P resulted in complete collapse of the PMF. Propidium iodide (PI) is a chromosome counter of red fluorescence staining of dead or dying cells with compromised membrane. The fluorescence is enhanced when the DNA of damaged cells is stained with PI. We further analyzed whether the L. monocytogenes cell membrane was disrupted by acidicin P using PI staining or not. As shown in Fig. 6C, higher fluorescence was observed in the cells of the acidicin P- and nisin-treated group than in the untreated cells. The increased fluorescence caused by acidicin P appeared concentration dependent. These results indicate that acidicin P increased the permeability and disrupted the cell membrane of L. monocytogenes. The disruption of membrane permeability caused by acidicin P may perturb cellular ATP levels of bacterial cells. The acidicin P-treated group had significantly higher extracellular ATP (Fig. 6D) and lower intracellular ATP (Fig. 6E) than that of the control group. These results suggest that acidicin P caused the leakage of large molecules (ATP).

## DISCUSSION

In this study, we mined abundant unexploited two-component bacteriocin *in silico* and characterized a novel two-component bacteriocin, acidicin P, with broad-spectrum antibacterial activity by disrupting the cell envelope to control L. monocytogenes. We utilized the precursor peptides of 20 reported two-component bacteriocins to devise an *in silico* approach to identify potential two-component bacteriocins in sequenced microbial genomes. The results demonstrated the diversity of the identified putative two-component bacteriocin via SSN. The work presented here provided unprecedented insight into the poorly explored two-component bacteriocins. The previously reported two-component bacteriocin with antimicrobial or anti-inflammatory annotation in the SSN demonstrated the sequence feature of their group, allowing the prediction of novel two-component bacteriocin activity. In addition to providing insights into the diversity and distribution of this bacteriocin, establishing the known bacteriocins can highlight features likely to be associated with novel activity, thus evading the pitfalls of reinvestigating reported bacteriocins. Furthermore, large-scale analysis using similarity networks will facilitate future genomics-driven bioactive product discovery campaigns.

Genomic mining strategies have been used for exploring natural products in *Planomonospora* (15), Pseudomonas (16), and *Nocardia* (17). In our previous study, metagenomic mining was performed (14), and a novel two-component bacteriocin, acidicin P, was identified from *P. acidilactici*. The acidicin P contains a broad antibacterial spectrum inhibiting L. monocytogenes 1.10753, S. agalactiae 10465, enterotoxigenic E. coli K88, and vancomycin-resistant E. faecalis V583. One of the limitations of bacteriocin utilization is the narrow antibacterial spectrum. Nisin, the only commercially produced bacteriocin approved as a food additive by Food and Drug Administration (FDA), shows antibacterial activity only against Gram-positive bacteria, which restricts its application (18). However, acidicin P possesses antimicrobial activity against both Gram-positive and Gram-negative bacteria, indicating a wider applied range than nisin.

The results of time-kill kinetics analysis of acidicin P against L. monocytogenes reveal that the optimal activity was obtained when complementary peptides were present in approximately equal amounts.

Similarly, a study of lactococcin G also reported that equal amounts of two complementary peptides were necessary for obtaining bacteriocin activity, while the individual peptides are totally inactive (19). This may because genes encoding two peptides are adjacent to each other on the same transcriptional unit and therefore are transcribed to the same extent (20). The α-helix sequence and helicity are involved in a variety of functions, including cell localization, lipid interactions, and enhancing helix flexibility (21). They play important roles in the bactericidal activity of class IIa and class IIb bacteriocins. The results of CD spectra showed that each component of the bacteriocin acidicin P is unstructured in aqueous conditions, while it contains an intrinsic tendency to adopt a typical α-helical structure in 50% TFE (a natural structure inducer). The helicity is increased after their binding together compared to individual components of Adpβ. We suppose that Adpα and Adpβ interacted directly with each other by cell membrane-induced helix-helix binding to execute antimicrobial activity. This phenomenon has also been reported for plantaricin E/F and plantaricin J/K, in which α-helical structure was induced by 50% TFE (22).

The synergistic antimicrobial activity that was observed when combining two complementary peptides may be due to the two components interacting with each other and forming one functional unit to target cells. Two-component bacteriocins may form either a parallel or antiparallel transmembrane helical dimer based on the close contact of the GxxxG-like motifs in two peptides. The interaction model for lactococcin G suggests that LcnG-α and LcnG-β form a parallel transmembrane helix-helix structure upon the interaction between the G_7_xxxG_11_-motif (LcnG-α) and G_7_xxxG_11_-motif (LcnG-β) (23). In our study, the results of MD simulations suggest that the A_5_xxxG_9_ motif in Adpα and the S_16_xxxG_20_ motif in Adpβ interacted and enabled the two peptides to form an antiparallel transmembrane helical dimer. The antiparallel transmembrane structure contains more hydrogen bonds than the parallel structure, highlighting the stabilization in the helix-helix interaction and the close contact between the two peptides. Furthermore, there are several positively charged amino acids (arginine or lysine residues) in the C terminus of Adpα and the N terminus of Adpβ. These cationic amino acids would contact the negatively charged group at the head of the liposome to decrease the repulsion and be inserted into the cell membrane. There are two aromatic amino acids, F25 and F21, in Adpβ. It is reported that the aromatic amino acids appeared in the terminal region of peptides to stabilize the terminal region in the membrane interface (24). We suppose that F25 in the C terminus of Adpβ was on the outer part of the target cell membrane, while F21 was on the inner part as a space lock to stabilize acidicin P to the membrane-water interface. A study reported that the two-component peptides of plantaricin S were remained parallel when inserted into the bilayer (25). However, the interaction structure of acidicin P was changed with an angle between two peptides, which is different from previous findings. This may suggest that acidicin P interacts with a membrane protein, triggering a conformation alteration.

The interaction model of acidicin P was investigated through site-directed mutational analysis. The GxxxG motif or the GxxxG-like motif is usually found in the sequence of two-component bacteriocins.

A previous study reported that the G (in GxxxG-like motif) substituted with isoleucine in plantaricin S and lactococcin G caused a detrimental effect on antimicrobial activity (23, 25). This large hydrophobic residue may prevent the peptides from coming too close to each other. In our study, such a mutation of A5 and G9 in the A_5_xxxG_9_ motif (Adpα) and of S16 and G20 in the S_16_xxxG_20_ motif (Adpβ) substituted with isoleucine also caused a detrimental effect on antimicrobial activity, highlighting that GxxxG-like motifs are crucial for acidicin P to fight against targeted bacteria. Furthermore, the residues involved in forming hydrogen bonds were replaced with small residues of alanine (A). The antimicrobial activity of mutants decreased compared to that of the wild type, illustrating the importance of hydrogen bonds in helping acidicin P insert into the targeted cell membrane. These results further demonstrated that A5, N7, G9, and R14 in Adpα and D12, S16, R19, and G20 in Adpβ played a crucial role in stabilizing the helix-helix interaction of Adpα and Adpβ and were essential for the activity of acidicin P. The potential of genetic engineering techniques to modify the activity of bacteriocins and solve challenging problems in disease has been reported (26). Understanding the key residues which are important for the activity of acidicin P may help in genetically engineering acidicin P to enhance the activity and physiologic properties for a variety of biological applications.

The cell envelope of Gram-positive bacteria comprises the cell wall and the cytoplasmic membrane. MD simulation simulated a probable mode of action for acidicin P on the cytoplasmic membrane; however, the possible targets need to be identified further. We investigated the cell morphology and intracellular structure of acidicin P-treated L. monocytogenes by SEM and TEM, respectively. A previous study reported that the loss of cytoplasmic content, cytoplasmic membrane dissolution, and vacuolization were observed in bifidocin A-, plantaricin LPL-1-, and bacteriocin CHQS-treated cells (27–29). In our study, the declined cytoplasm density and vacuolization and the leaked cytoplasm around cell were also found in acidicin P-treated L. monocytogenes. This may be caused by the insertion of acidicin P into the cell membrane, which is supported by a previous report stating that inserting peptides into the lipid bilayer is an energy-costing process, which is likely offset by the formation of water- or ion-lined pores (21). A previous study reported that two-component bacteriocins, including lactococcin G, plantaricin E/F, plantaricin J/K, lactocin 705, thermophilin 13, and lactacin F, only target cell membranes against sensitive bacteria (12). However, in our study, severe wrinkles and a noticeable hollowness in the cell wall were observed, indicating that the cell wall of L. monocytogenes was also destroyed by acidicin P. Therefore, we suppose that acidicin P destroyed the cell envelope of L. monocytogenes.

Despite the function of cell morphology in maintaining the cell wall, it is not a powerful barrier to exclude antibiotics because of the pores in it (30). However, the cytoplasmic membrane is a selectively permeable barrier which prevents intracellular content leakage and plays a critical role in cellular activities, including transportation of substances, cellular respiration, and cell-cell communication (31, 32). Therefore, the disruption of acidicin P on the bacterial cytoplasmic membrane was investigated. The PMF is the electrochemical gradient of protons caused by the extrusion of protons generated across the cell membrane. It is essential for several critical bacterial processes, such as ATP synthesis, protein excretion, and flagellar motility. The PMF is composed of the transmembrane proton gradient (ΔpH) and electric potential (Δφ) to maintain homeostasis. Disruption of any component will cause a compensatory increase in the other one to maintain a constant value for the PMF. The cooperative dissipation of ΔpH and Δφ induced collapse of the PMF, which results in membrane energetic disruption and the loss of bacterial viability (33). DiSC_3_(5) is a popular probe to study the membrane potential. It accumulates in the cytoplasmic membrane and is released into the medium, causing fluorescence to increase when Δφ is disrupted, whereas the fluorescence decreases when ΔpH is disrupted (33). We analyzed the PMF of L. monocytogenes cells treated with acidicin P using DISC_3_(5). Nisin was used as a positive control in our study, which has been reported to induce depolarization of the cytoplasmic membrane and efflux of cellular ATP of L. monocytogenes (34). In our experiments, lower concentrations of acidicin P (<6.25 μg/mL) disrupted PMF transitorily, possibly due to either the ΔpH or Δφ of the L. monocytogenes cell membrane being disrupted and causing a compensatory change to the other. However, higher concentrations (12.5 to 25 μg/mL) of acidicin P resulted in complete collapse of the PMF, possibly because acidicin P caused immediate disruption of Δφ that could not be compensated for by ΔpH. A higher concentration of acidicin P causes PMF disruption of target cells, similar to other bacteriocins, such plantaricin NC8, which may fight against Micrococcus luteus (35), and lysostaphin, which inhibits Staphylococcus aureus (36). The effect of acidicin P on the physical integrity of the L. monocytogenes cytoplasmic membrane was assessed using the red fluorescent nuclear dye PI, which labels cells with a compromised membrane. Nisin was used as a positive control in our study, which can form transient multistate pores on target cell membranes to inhibit or kill their target bacterium (9). Our results found that acidicin P caused increased fluorescence, indicating that acidicin P disrupted the cytoplasmic membrane integrity of L. monocytogenes like nisin did. The disruption of membrane permeability caused by acidicin P may perturb the cellular ATP levels of bacterial cells. The ATP plays a key role in many metabolic processes, and the efflux of ATP will induce serious damage to cells (37). In our experiment, the acidicin P declined the level of intracellular ATP and increased the extracellular ATP level, which correlated with the result of PI uptake and the vacuolization observed in TEM, in line with the leakage assay of the cell constituents in L. monocytogenes caused by the class IIa bacteriocin plantaricin LPL-1 (27). Therefore, we speculate that acidicin P fights against L. monocytogenes cells by disrupting cytoplasm membrane integrity and targeting cytoplasm membrane energetics.

**FIG 7:**
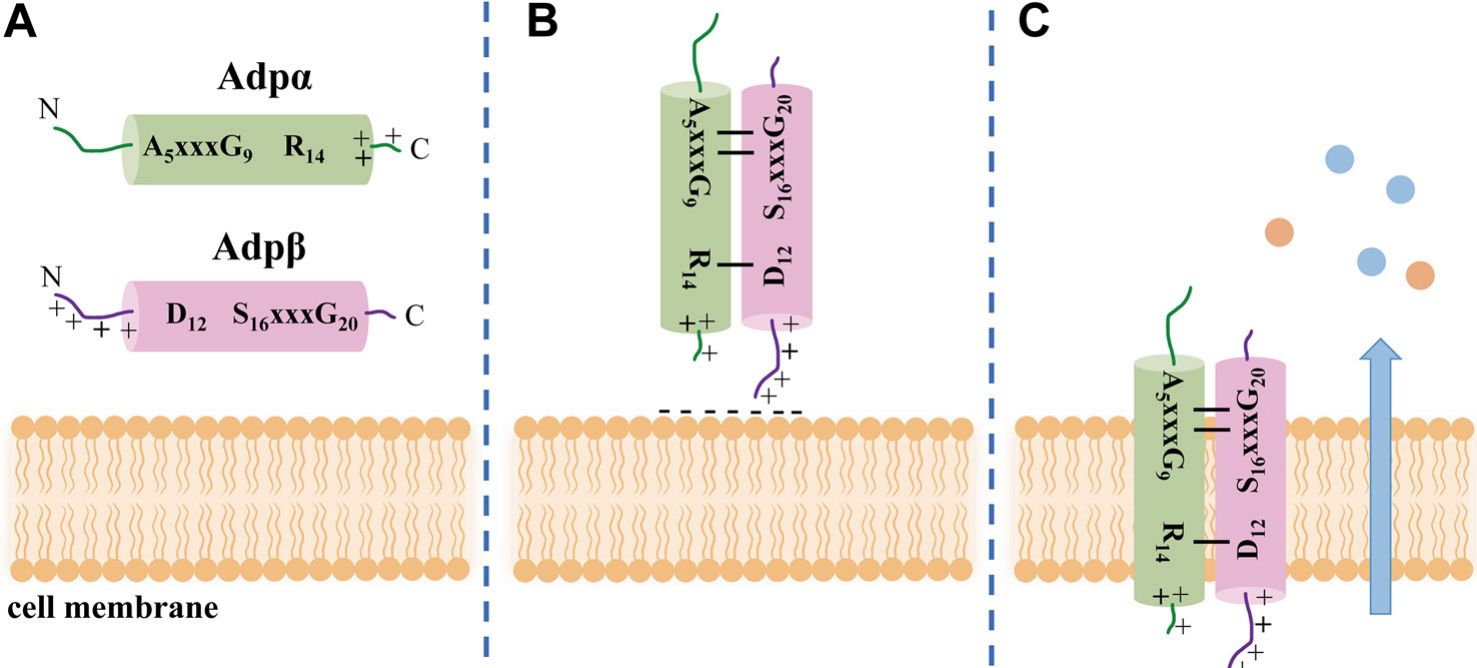
Schematic diagram of the biological activity of acidicin P. (A) Helical structure of Adpα and Adpβ in the membrane environment. +, the cationic amino acid. (B) Antiparallel helical dimer of acidicin P. The lines between Adpα and Adpβ represent hydrogen bonds. (C) Structure of acidicin P being inserted into the cell membrane which positions the cationic region within the target cell.

**Conclusion.** Acidicin P is the first two-component bacteriocin reported in *P. acidilactici* and belongs to an unusual group of two-component bacteriocins. The two components, Adpα and Adpβ, form an antiparallel helix-helix structure due to the specific recognition between the A_5_xxxG_9_ motif of Adpα and the S_16_xxxG_20_ motif of Adpβ. The hydrogen bonding between the two peptides maintained close contact with each other and played a key role in the antimicrobial activity of acidicin P (Fig. 7A and B). Acidicin P is irreversibly inserted into the cell membrane lipid bilayer, causing severe permeabilization and damaging the cell morphology and ultrastructure to kill L. monocytogenes (Fig. 7C). Acidicin P has the potential to be applied as an antilisterial drug.

## MATERIALS AND METHODS

### Bacterial strains and synthesis of peptides.

The bacteriocin producer *P. acidilactici* LAC5-17 was isolated from Chinese spicy cabbage using metagenomic mining of isolates population (MMIP) method in our previous work (14). The biosynthetic gene cluster information of acidicin P was obtained from the metagenomic data of our previous work (14).

Adpα, Adpβ and their site-directed mutants were obtained by solid phase peptide synthesis (25). The purity of the peptides was verified to be greater than 95% by analytical reversed-phase high-pressure liquid chromatography (HPLC). The desired product was confirmed by electrospray ionization mass spectrometry (ESI-MS).

### Bacteriocin activity assay.

The antimicrobial spectrum was investigated using the indicator strains Streptococcus agalactiae 10465, Listeria monocytogenes 1.10753, enterotoxigenic Escherichia coli K88, vancomycin-resistant Enterococcus faecalis V583, Salmonella enterica serovar Typhimurium 14028, methicillin-resistant Staphylococcus aureus, and Bacillus cereus 1.1846. L. monocytogenes 1.10753 was used for growth curve measurement with a microtiter plate assay system. The indicator (at 1 × 10^5^ CFU/mL) and the peptide (at 2-fold dilutions) were added to a final volume of 200 μL in each well. The bacterial growth was monitored with Bioscreen C (OyGrowth Curves Ab Ltd.) at 600 nm at 30°C. The MIC was defined as the total amount of Adpα, Adpβ, and acidicin P (the complementary peptides combined with equal amounts) that inhibited the growth of the indicator strain. The agar well diffusion method was used to investigate the antimicrobial activity of the mutations. The L. monocytogenes was inoculated in a brain heart infusion (BHI) agar plate as an indicator at a concentration of 10^5^ CFU/mL. The peptide mutants (40 μL) were added to the wells (5 mm), and the plates were incubated for 24 h at 30°C.

### Circular dichroism (CD) spectroscopy.

Peptides were prepared in either aqueous buffer or 50% trifluoroethanol (TFE) at a concentration of 0.2 mg/mL. The CD spectra were recorded with a JASCO 715 spectropolarimeter. Data were collected every 1 nm from 180 to 260 nm. All measurements were conducted at least twice using a 1-mm cuvette.

### Modeling and molecular dynamics (MD) simulation methods.

Sequence alignment between acidicin P and other proteins with reported structures was performed. No crystal structure was found corresponding to Adpα and Adpβ in the RCSB Protein Data Bank (PDB) database. However, Adpβ contained a high sequence similarity (56%) to protein Plsβ (PDB: 6GO0). Therefore, the three-dimensional structure of Adpβ was determined by homology modeling using Modeler, and the structure of Adpα was obtained by molecular dynamics simulation. Then, the structure of the dimer formed by Adpα and Adpβ (in an antiparallel or parallel orientation) was calculated using Rosetta. In total, 100 structures were calculated, and the lowest docking energy structure was obtained as a dimer model for the MD simulations. The cell membrane of Gram-positive bacteria was mimicked by Charmm-GUI with 1-palmitoyl-2-oleoyl-sn-glycero-3-phosphoglycerol (POPG) and 1-palmitoyl-2-oleoyl-sn-glycero-3- phosphoethanolamine (POPE) in a ratio of 3:1. The dimer was placed above the model membrane at a distance of 4.3 nm. The MD simulation was performed using Amber under constant pressure and temperature. The full MD simulation was carried out for 500 ns.

### Proton motive force (PMF) assay.

L. monocytogenes was incubated in BHI broth at 37°C to the exponential phase (optical density at 600 nm [OD], 0.6). Then the medium was centrifuged and the supernatant was discarded. The cell precipitation was washed twice with phosphatic buffer solution (PBS). Finally, the cells were resuspended in 1 mL PBS and incubated with 1 μM membrane potential-sensitive dye 3,3-dipropylthiadicarbocyanine iodide [DiSC_3_(5), AAT Bioquest] at 37°C for 10 min. Then, the incubated cells were treated with different concentrations of acidicin P. Measurements were performed with a Synergy H4 device (BioTek) at 30°C with 622 nm excitation and 670-nm emission filters. The cells treated with nisin Z (Zhejiang Silver-Elephant Bic-engineering Co., Ltd.) and PBS were designed as a positive control and negative control, respectively.

### Cytoplasmic membrane permeability assay.

Pretreatment of L. monocytogenes cells for the cytoplasmic membrane permeability assay was similar to that for the PMF assay. The cells were resuspended in 1 mL PBS and treated with different concentrations of acidicin P at 30°C for 30 min. The treated cells were stained with 10 μg/mL propidium iodide (PI, Bioss) in the dark for 15 min. Emission spectra of the mixture were recorded at 615 nm upon excitation at 535 nm with a Synergy H4 device (BioTek).

### ATP assay.

Pretreatment of L. monocytogenes cells for the ATP assay was similar to that for the PMF assay. The cells were treated with different concentrations of acidicin P at 30°C for 30 min. Then, the mixture was centrifuged at 13,000 rpm for 5 min at 4°C. The supernatant was collected for the measurement of extracellular ATP according to the instructions of the ATP assay kit (Beyotime Bioengineering Institute). Then, the cell pellet was added with lysis buffer and disrupted ultrasonically for 1 min. The supernatant was collected for intracellular ATP concentration analysis.

### Scanning electron microscopy (SEM).

Pretreatment of L. monocytogenes cells for SEM was similar to that for the PMF assay. L. monocytogenes cells were treated with acidicin P at final concentrations of 6.25 μg/mL and 12.5 μg/mL at 30°C for 30 min. The cells were washed with phosphatic buffer solution (PBS) and fixed with 2.5% glutaraldehyde at 4°C for 8 h. Subsequently, the cells were dehydrated gradually with ethanol (30%, 50%, 70%, 80%, 90%, and 100%) and coated with gold-palladium for SEM (Hitachi-SU8010) analysis.

### Transmission electron microscopy (TEM).

Pretreatment of L. monocytogenes cells for TEM was similar to that for SEM. The cells were fixed with 2.5% glutaraldehyde at 4°C for 1 h and postfixed with 1% osmic acid for 1 h in the dark. Subsequently, the cells were dehydrated with ascending concentrations of acetone (30%, 50%, 70%, 85%, 95%, and 100%), embedded using resin (LR White, Sigma), and sliced for TEM (JEM-1400) analysis. The L. monocytogenes cells were treated with PBS as a control group.

### Bioinformatic analysis.

Previously reported precursor peptides of two-component bacteriocins were used as query sequences for PSI-BLAST with default search parameters. The sequence was analyzed by EFI-EST (https://efi.igb.illinois.edu/efi-est/) to generate the sequence similarity network (SSN). Then, the SSN was visualized using Cytoscape 7.0.2.

### Statistical analysis.

All experiments were done at least three times. Data were analyzed for analysis of variance (ANOVA) using GraphPad Prism 8 to determine the least significant differences (*P *< 0.05). The results are presented as the mean ± standard deviation (SD).
