# Supplementary material for: A Novel Two-Component Bacteriocin, Acidicin P, and Its Key Residues for Inhibiting Listeria monocytogenes by Targeting the Cell Membrane
Source: Microbiol Spectr. 2023 Jun 8;11(4):e05210-22. doi: 10.1128/spectrum.05210-22 (PMC10434283; doi:10.1128/spectrum.05210-22)
Supplement: Supplemental file 1 — Table S1. Download spectrum.05210-22-s0001.pdf, PDF file, 0.2 MB [file spectrum.05210-22-s0001.pdf]

Table S1 Sequence of two-component bacteriocins

Bacteriocins	Sequences	Activity	Reference
**ABP-118**	Abp118α: KRGPNCVGNFLGGLFAGAAAGVPLGPAGIVGGANLGMVGGALTCL	*L. monocytogenes*	(2)
	Abp118β: KNGYGGSGNRWVHCGAGIVGGALIGAIGGPWSAVAGGISGGFTSCR		
**Amylovorin L**	amyLα: SKGKGRNNWAGNTIGIVSSAATGAALGSAICGPGCGFVGAHWGAVGWTAVASFSGAFGKIRK		(2)
	amyLβ: NRWTNAYSAALGCAVPGVKYGKKLGGVWGAVIGGVGGAAVCGLAGYVRKG		
**Acidocin LF221B**	LF221Bα: NNVNWGSVAGSCGKGAVMEIYFGNPILGCANGAATSLVLQTASGIYKNYQKKR	*Clostridium sp.*	(2)
	LF221β: NKWGNAVIGAATGATRGVSWCRGFGPWGMTACALGGAAIGGYLGYKSN		
**Brochocin-C**	BrcA: YSSKDCLKDIGKGIGAGTVAGAAGGGLAAGLGAIPGAFVGAHFGVIGGSAACIGGLLGN	*Salmonella*	(1)
	BrcB: KINWGNVGGSCVGGAVIGGALGGLGGAGGGCITGAIGSIWDQW	*Typhimurium*	
**Carnobacteriocin X**	CbnX: WGWKEVVQNGQTIFSAGQKLGNMVGKIVPLPFG		(2)
	CbnY: SAILAITLGIFATGYGMGVQKAINDRRKK		
**Enterocin 1071**	Entα: ESVFSKIGNAVGPAAYWILKGLGNMSDVNQADRINRKKH		(2)
	Entβ: GPGKWLPWLQPAYDFVTGLAKGIGKEGNKNKWKNV		
**Enterocin X**	enterocin Xα: SNDSLWYGVGQFMGKQANCITNHPVKHMIIPGYCLSKILG	*L. innocua*	(2)
	enterocin Xβ: IAPIIVAGLGYLVKDAWDHSDQIISGFKKGWNGGRRK		
**Gassericin S**	GasA: KNWSVAKCGGTIGTNIAIGAWRGARAGSFFGQPVSVGAGALIGASAGAIGGSVQCVGWLAGGGR		(2)
	GasX: NNVNWGSVAGSCGKGAVMGIYFGNPILGCANGAATSLVLQTTSGIYKNYQKKR		
**Gassericin T**	GatA: RNNWAANIGGVGGATVAGWALGNAVCGPACGFVGAHYVPIAWAGVTAATGGFGKIRK		(2)
	GatX: NKWGNAVIGAATGATRGVSWCRGFGPWGMTACGLGGAAIGGYLGYKSN		
**Lactocin 705**	705α: GMSGYIQGIPDFLKGYLHGISAANKHKKGRLGY		(2)
	705β: GFWGGLGYIAGRVGAAYGHAQASANNHHSPING		
**Lactacin F**	LafA: RNNWQTNVGGAVGSAMIGATVGGTICGPACAVAGAHYLPILWTGVTAATGGFGKIRK		(1)
	LafX: NRWGDTVLSAASGAGTGIKACKSFGPWGMAICGVGGAAIGGYFGYTHN		
**Lactococcin G**	LcnG-α: GTWDDIGQGIGRVAYWVGKAMGNMSDVNQASRINRKKKH		(2)

	LcnG-β: KKWGWLAWVDPAYEFIKGFGKGAIKEGNKDKWKNI		
**Lactococcin Q**	LcnQα: SIWGDIGQGVGKAAYWVGKAMGNMSDVNQASRINRKKKH		(2)
	LcnQβ: KKWGWLAWVEPAGEFLKGFGKGAIKEGNKDKWKNI		
**Mutacin IV**	NlmA: KVSGGEAVAAIGICATASAAIGGLAGATLVTPYCVGTWGLIRSH		(2)
	NlmB: DKQAADTFLSAVGGAASGFTYCASNGVWHPYILAGCAGVGAVGSVVFPH		
**Plantaricin E/F**	PlnE: FNRGGYNFGKSVRHVVDAIGSVAGIRGILKSIR	*Escherichia coli K1.1*	(2)
	PlnF: VFHAYSARGVRNNYKSAVGPADWVISAVRGFIHG		
**Plantaricin J/K**	PlnJ: GAWKNFWSSLRKGFYDGEAGRAIRR	*Candida albicans*	(2)
	PlnK: RRSRKNGIGYAIGYAFGAVERAVLGGSRDYNK		
**Plantaricin S**	PlSα: RNKLAYNMGHYAGKATIFGLAAWALLA	*L. monocytogenes,*	(2)
	PlSβ: KKKKQSWYAAAGDAIVSFGEGFLNAW	*Enterococcus faecalis*	
**Plantaricin NC8**	PLNC8α: DLTTKLWSSWGYYLGKKARWNLKHPYVQF	*Staphylococcus spp.*	(2)
	PLNC8β: SVPTSVYTLGIKILWSAYKHRKTIEKSFNKGFYH	Anti-inflammatory	
**Salivaricin P**	Slnα: KRGPNCVGNFLGGLFAGAAAGVPLGPAGIVGGANLGMVGGALTCL	*L. monocytogenes*	(2)
	Slnβ: KNGYGGSGNRWVHCGAGIVGGALIGAIGGPWSAVAGGISGGFASCH		
**Thermophilin 13**	ThmA: YSGKDCLKDMGGYALAGAGSGALWGAPAGGVGALPGAFVGAHVGAIAGGFACMGGMIGNKFN		(2)
	ThmB: QINWGSVVGHCIGGAIIGGAFSGGAAAGVGCLVGSGKAIINGL		

References

1. Nissen-Meyer J, Oppegard C, Rogne P, Haugen HS, Kristiansen PE. 2010. Structure and Mode-of-Action of the Two-Peptide (Class-IIb) Bacteriocins. Probiotics Antimicro 2: 52-60.
2. Yi YL, Li P, Zhao F, Zhang TT, Shan YY, Wang X, Lu X. 2022. Current status and potentiality of class II bacteriocins from lactic acid bacteria: structure, mode of action and applications in the food industry. Trends Food Sci Tech 120: 387-401.
